# Supplementary material for: Comprehensive benchmarking and guidelines of mosaic variant calling strategies
Source: Nat Methods. 2023 Oct 12;20(12):2058–67. doi: 10.1038/s41592-023-02043-2 (PMC10703685; doi:10.1038/s41592-023-02043-2)
Supplement: Supplementary file 1 — Supplementary Figs. 1–26, Notes 1–10 and Tables 6–12. [file 41592_2023_2043_MOESM1_ESM.pdf]

nature methods

Analysis

https://doi.org/10.1038/s41592-023-02043-2

Comprehensive benchmarking and guidelines of mosaic variant calling strategies

In the format provided by the authors and unedited

Supplementary Figures

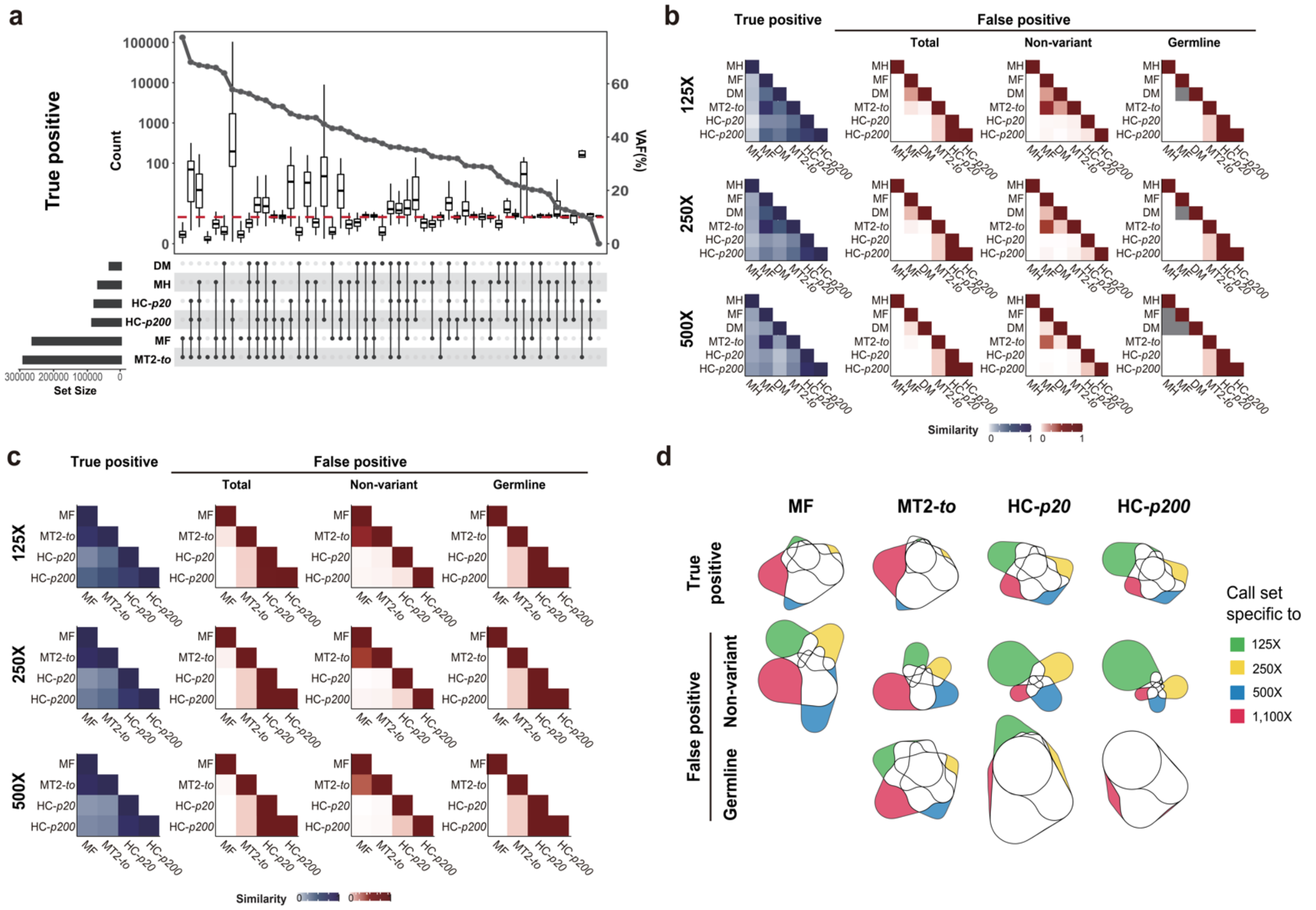

Supplementary Figure 1. Evaluation on call set consistency and similarity of variant detection in single sample (a-b) Similarity of call sets are between different detection approaches are shown in lower depths (125×, 250×, and 500×), calculated with Jaccard index. **(a)** Similarity of SNV call sets. **(b)** Similarity of INDEL call sets. **(c)** The compositions of the true positives from the evaluated detection approaches are shown. The count of intersections of the call sets and VAF distribution within each are shown with the left- and right-y axis respectively. Red dashed line refers to 10% of VAF and the median and quantiles are shown by the boxes with minima and maxima as whiskers. 39 truth sets in 1,100× depth were utilized for the analysis. **(d)** Euler diagrams illustrating the consistency of the INDEL call sets for single sample analysis towards four different sequencing depth (125×, 250×, 500×, and 1,100×), 39 truth sets utilized for each depth. True positives and two types of false positives (non-variant and germline variant) are represented based on the relative sizes and relationships between the call sets are shown. Colored parts represent the call set specific to each depth (nVennR).

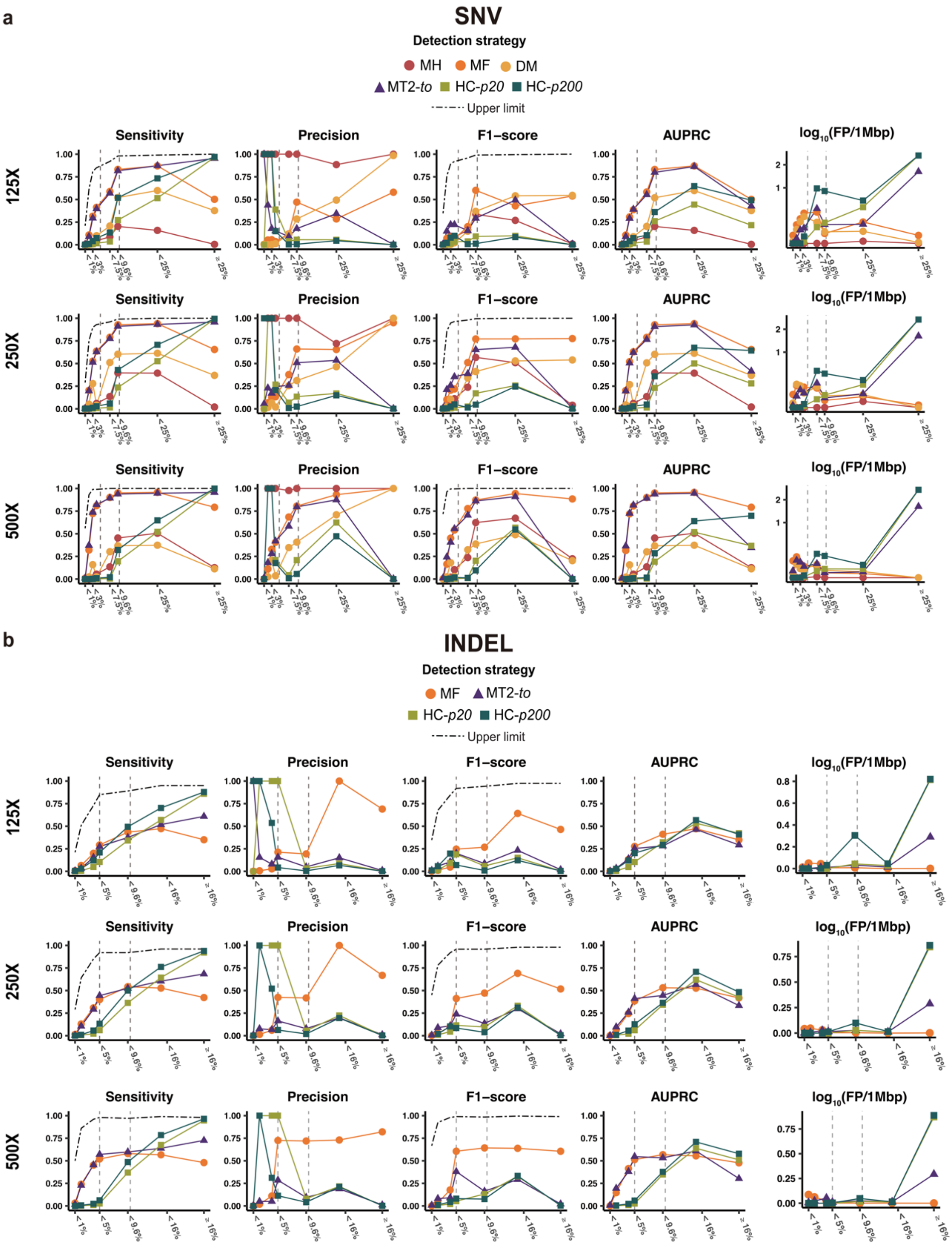

Supplementary Figure 2. Performance evaluation in single sample (a-b) Mosaic SNV and INDEL detection performance was evaluated on three different sequencing depth (125X, 250X, and 500X) with 39 truth sets. Sensitivity, precision, F1-score, AUPRC, and the false positive rate ($\log_{10}$FP/1Mbp) in different VAF bins categories are shown. Dashed line with dots depicts the theoretical upper limit for sensitivity and F1-score owing to the depletion of mutant alleles in sequencing data at low-VAF. The two vertical lines with gray dash refers to VAF 5% and 10%. The y-axis of $\log_{10}$(FP rate) is shown in square root. **(a)** Evaluation on SNV calling performance in 8 bins (<1%, 1%–2%, 2%–3%, 3%–4%, 4%–7.5%, 7.5%–9.6%, 9.6%–25%, and ≥ 25%). **(b)** Evaluation on INDEL calling performance in 7 bins (<1%, 1%–2%, 2%–4%, 4%–5%, 5%–9.6%, 9.6%–16%, and ≥ 16%).

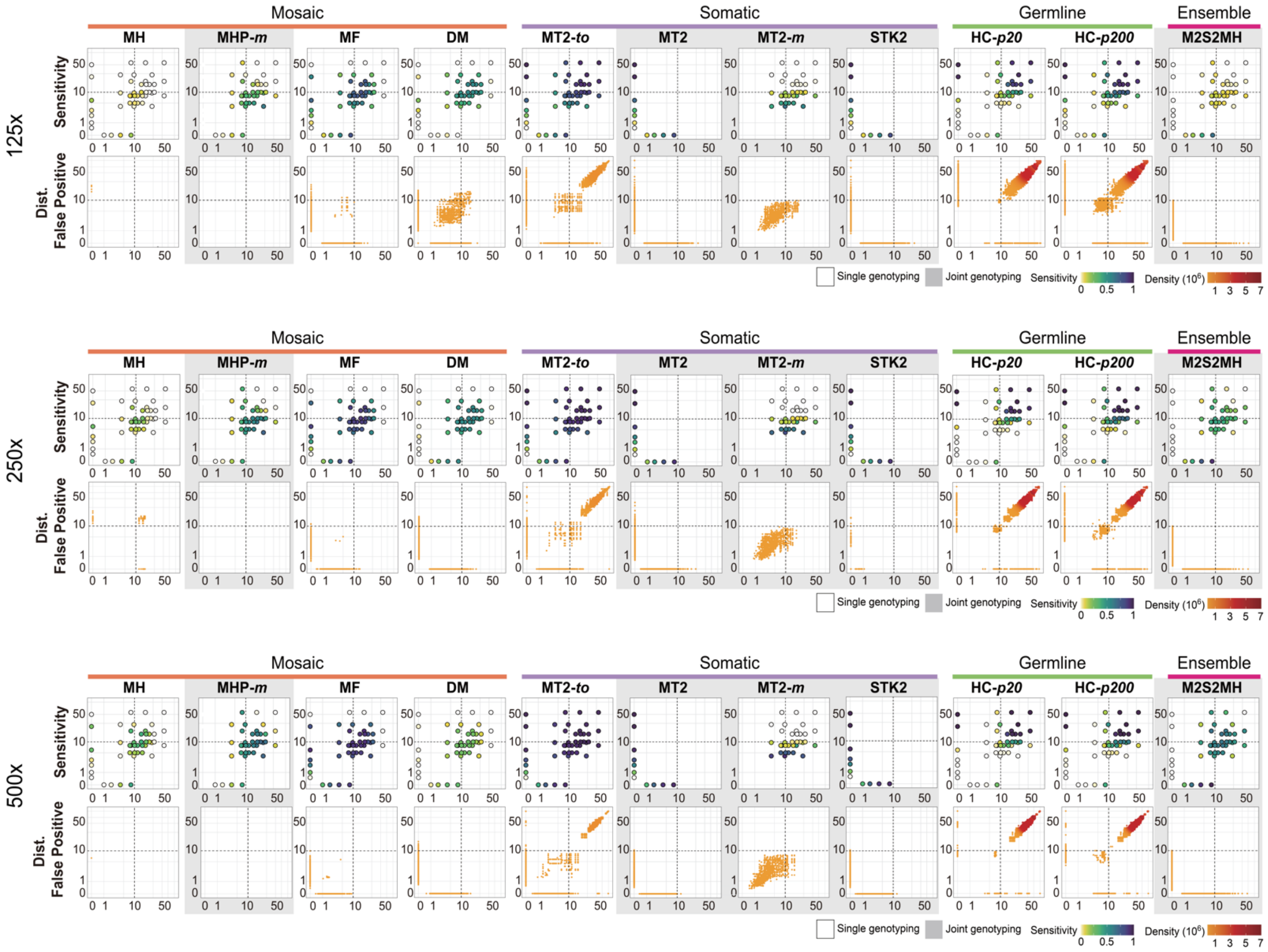

Supplementary Figure 3. Evaluation on SNV detection in paired-sample. Sensitivity and distribution of false positives of the eleven evaluated approaches for SNV detection are shown for three different sequencing depths (125X, 250X, and 500X), by using 39 truth sets. Sensitivities in all possible combinations of expected VAF pairs were binned and shown as heat maps within each circle on the plane and axis. Points at the plane and x or y axis refers to shared and sample-specific variants, respectively. Observations along VAFs are shown on a $\log_{10}$ scale and the dashed line refers to 10%. Callers within gray box used joint genotyping.

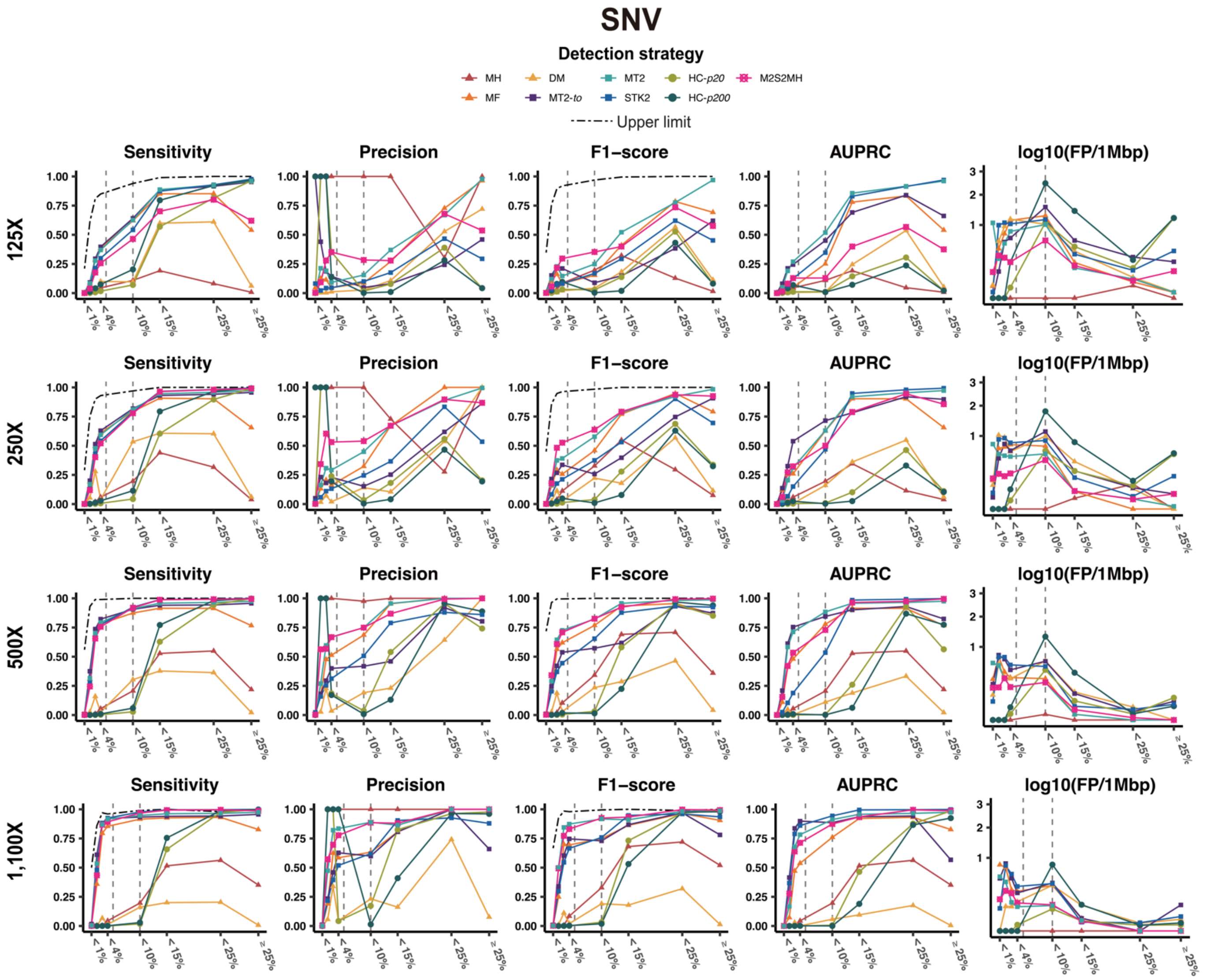

Supplementary Figure 4. Evaluation on sample-specific SNV detection in paired-sample. Sensitivity, precision, F1-score, AUPRC , and the false positive rate ($\log_{10}$FP/1Mbp) in different VAF bins categories are shown in four different sequencing depths (125×, 250×, 500×, and 1,100×). 356 combinations of sample pairs were tested by taking eighteen M3 truth sets as the case sample and twenty-one M1 and M2 (9 M1 and 12 M2) sets as controls. Performance is shown in 8 VAF bins (<1%, 1%–2%, 2%–3%, 3%–4%, 4%–10%, 10%–15%, 15%–25%, and ≥25%) and dashed lines point to 5% and 10%. Dashed line with dots depicts the theoretical upper limit for sensitivity and F1-score owing to the depletion of mutant alleles in sequencing data at low-VAF (**Supplementary Notes**). The two vertical lines with gray dash refers to VAF 5% and 10%. The y-axis of $\log_{10}$(FP rate) is shown in square root.

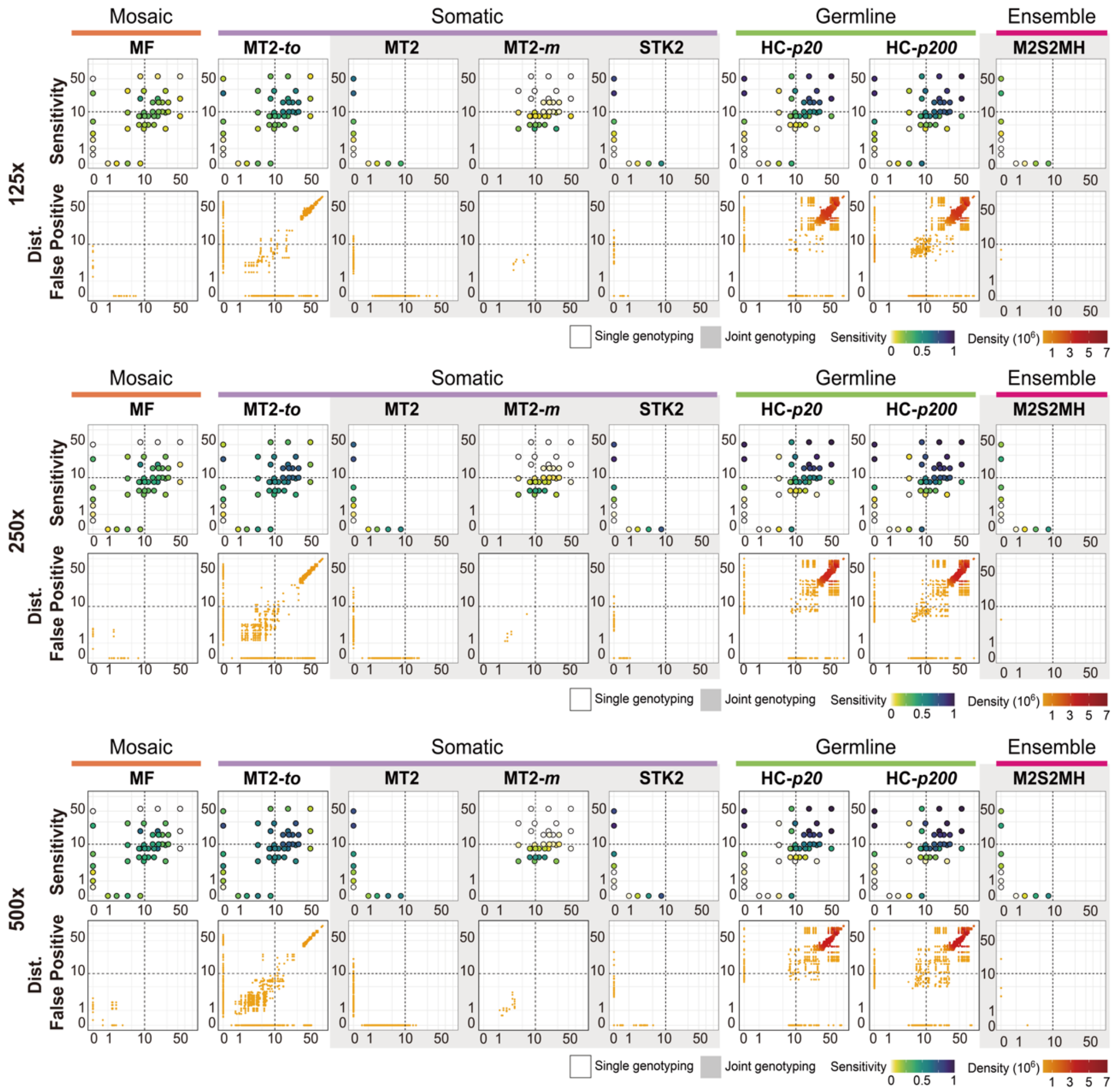

Supplementary figure 5. Evaluation on INDEL detection in paired sample Sensitivity and distribution of false positives of the eight evaluated approaches for INDEL detection are shown for three different sequencing depths (125X, 250X, and 500X), by using 39 truth sets. Sensitivities in all possible combinations of expected VAF pairs were binned and shown as heat maps within each circle on the plane and axis. Points at the plane and x or y axis refers to shared and sample-specific variants, respectively. Observations along VAFs are shown on a $\log_{10}$ scale and the dashed line refers to 10%. Callers within gray box used joint genotyping.

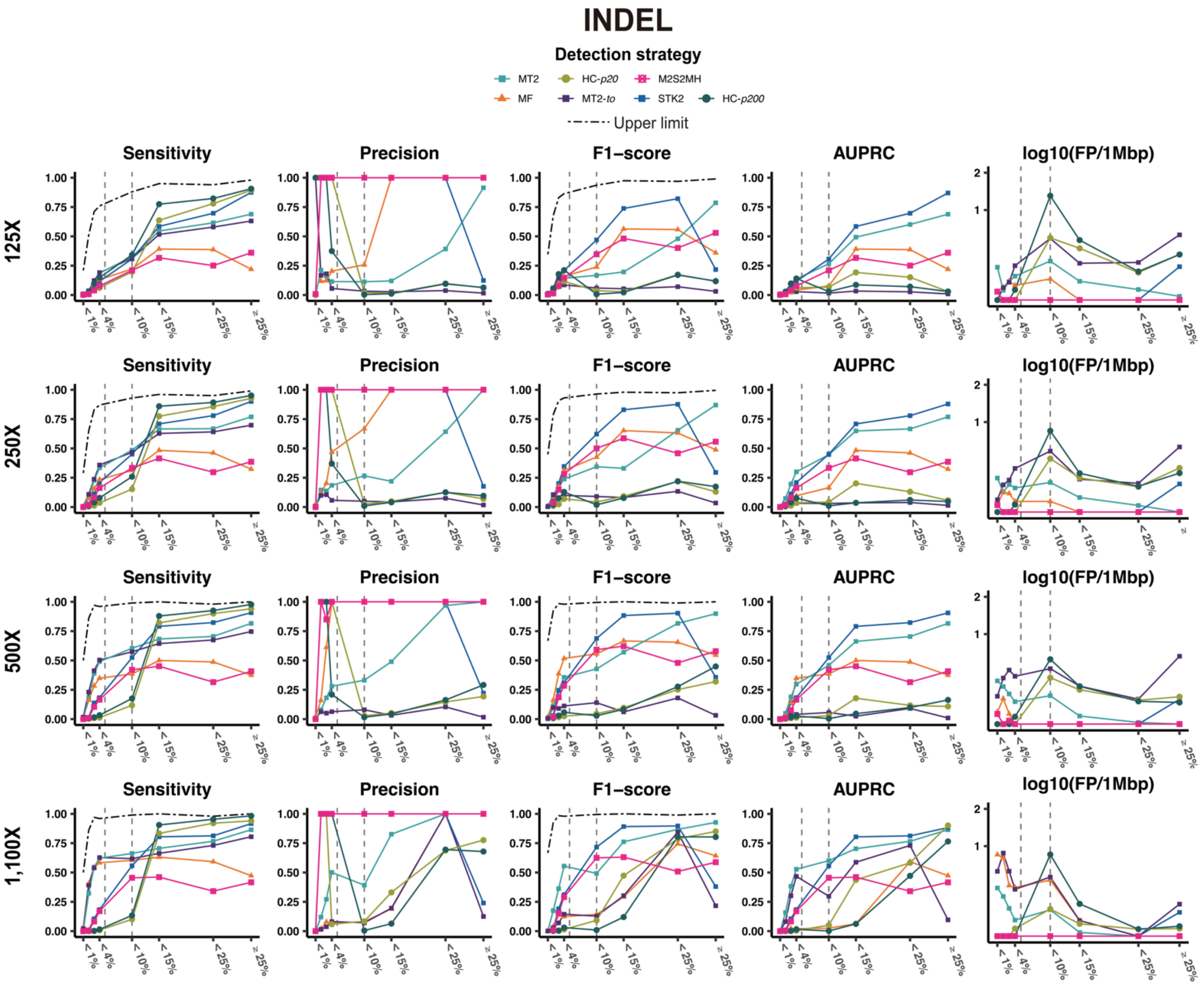

Supplementary Figure 6. Evaluation on sample-specific INDEL detection in paired-sample (a-b) Sensitivity, precision, F1-score, and the false positive rate (log_{10}FP/1Mbp) in different VAF bins categories are shown in four different sequencing depths (125×, 250×, 500×, and 1,100×). 356 combinations of sample pairs were tested by taking eighteen M3 truth sets as the case sample and twenty-one M1 and M2 (9 M1 and 12 M2) sets as controls. Performance is shown in 8 VAF bins (<1%, 1%–2%, 2%–3%, 3%–4%, 4%–10%, 10%–15%, 15%–25%, and ≥25%) and dashed lines point to 5% and 10%. Dashed line with dots depicts the theoretical upper limit for sensitivity and F1-score owing to the depletion of mutant alleles in sequencing data at low-VAF (**Supplementary Notes**). The two vertical lines with gray dash refers to VAF 5% and 10%. The y-axis of log_{10}(FP rate) is shown in square root.

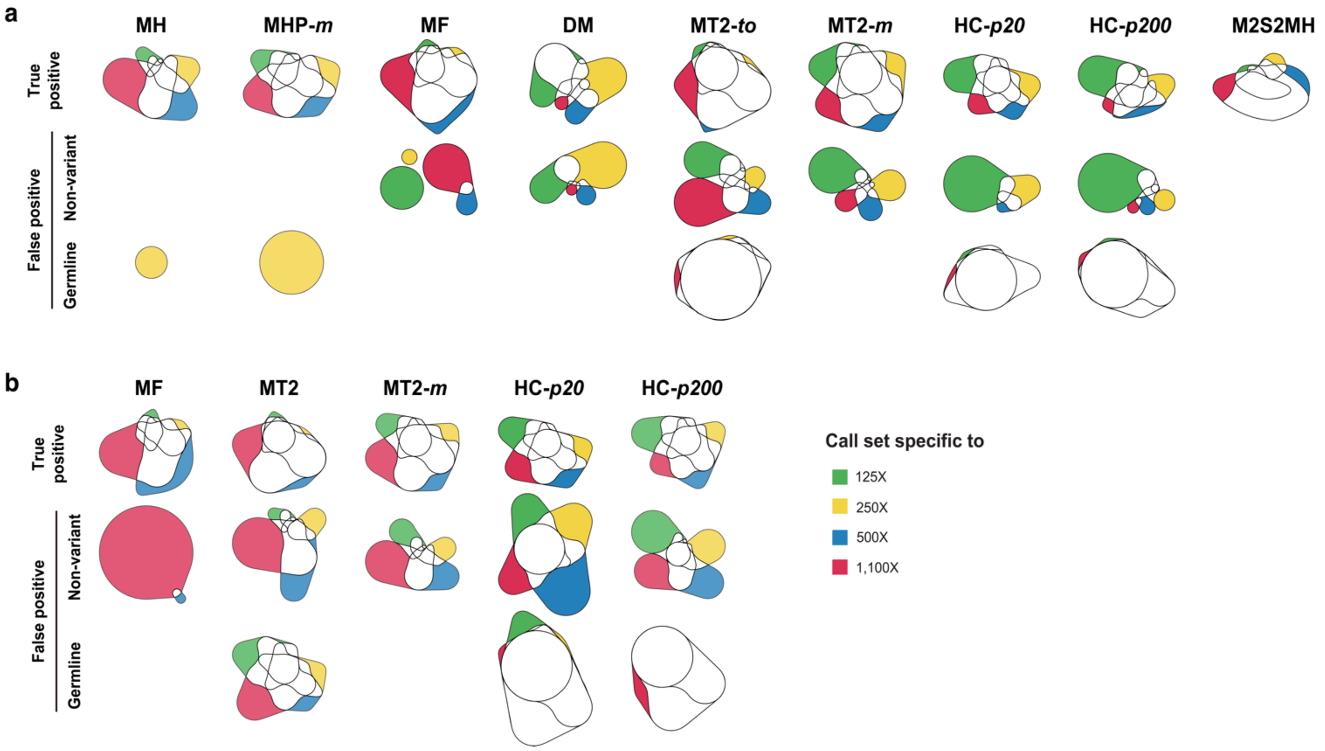

Supplementary Figure 7. **Evaluation on call set consistency and similarity of shared variant detection in paired-sample** (a-b) Euler diagrams illustrating the consistency towards four different sequencing depths (125×, 250×, 500×, and 1,100×) of the shared SNV and INDEL call sets. True positives and two types of false positives (non-variant and germline variant) are represented based on the relative sizes and relationships between the call sets, calculated with Jaccard index. Colored parts represent the call set specific to each depth (nVennR) (a) shared SNV (b) shared INDEL

Supplementary Notes

Table of contents

[1] Selection of ploidy in HaplotypeCaller and the ploidy effect 9

[2] The theoretical upper limits in performance evaluation 10

[4] Defining the relative distance between samples in a developmental lineage tree 12

[5] The effect of capture bias in the exome-based benchmark result 13

[6] Possible noise in the ground truth data 15

[7] The impact of the populational frequency database utilization with germline variant-derived ground truth data 16

[8] Performance evaluation with biological datasets and confirmation of the suggested recommendation 17

(1) VAF landscapes of the mosaic variants in the ground truth and the biological datasets 19
(2) Mosaic variant detection in a single sample 19
(3) Call set and feature level recombination in mosaic calling 21
(4) Sample-specific mosaic variant detection in paired-sample 22
(5) Shared mosaic variant detection in paired-sample 23

[9] Integrity of the cell lines utilized for the reference standard 27

[10] Selection of the raw input for MosaicForecast 27

[1] Selection of ploidy in HaplotypeCaller and the ploidy effect

We chose to use ploidy 20 and ploidy 200 in this benchmark based on the recommendation of a previous paper from Brain Somatic Mosaicism Network (BSMN)[1], which sets the ploidy to the 20% of the overall sequencing coverage. Therefore, we chose ploidy 20 and 200 in our evaluation, as our benchmark data ranged from 125× to 1,100×. We additionally tested two different ploidy settings, ploidy 50 and 100, to inspect the effect of the ploidy values on the mosaic SNV detection performance. We found a clear trade-off between sensitivity and specificity. The higher ploidy option increased sensitivity but lost precision, and vice versa in the lower ploidy options (**Supplementary Figure 8**). But the overall classification power (F1-score) remained similar. Therefore, ploidy options can be selected lower or higher from the 20% of the overall coverage depending on the research purposes.

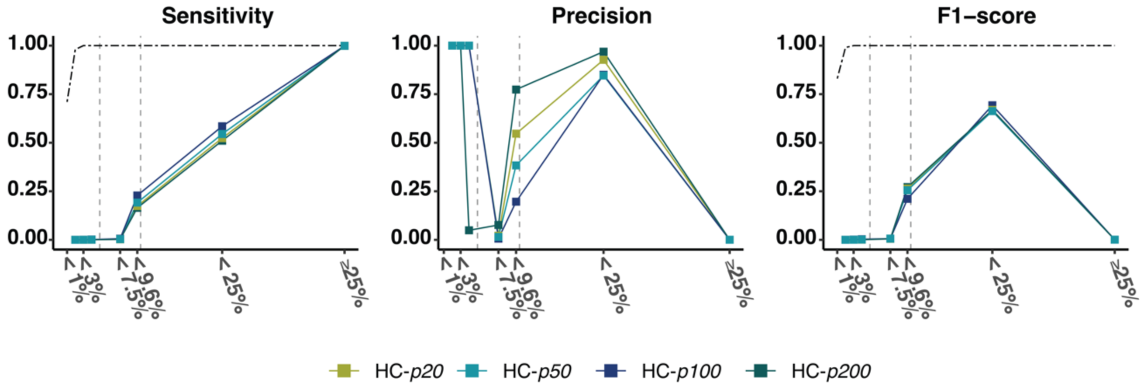

Supplementary Figure 8. Evaluation on ploidy settings of HaplotypeCaller in single sample analysis. The SNV detection performance was tested with 1,100× data of all 39 pairs of ground truth data in eight VAF bins (<1%, 1%–2%, 2%–3%, 3%–4%, 4%–7.5%, 7.5%–9.6%, 9.6%–25%, and ≥ 25%). Dashed lines with dots depict the upper limit for the true positives. The two vertical lines with gray dash refers to VAF 5% and 10%.

[2] The theoretical upper limits in performance evaluation

In performance evaluation, there can exist two types of false negatives (FN), (1) in which the true alternative alleles are present in the data or (2) the alternative alleles are absent in data. In the case of (2), the undetected sites are not truly false negatives that come from variant calling performance and should be distinguished from (1).
To first investigate the positions of (2), we re-assessed the VAFs of the positions in the original cell line data to check if there were sufficient allele frequencies to be germline variants. For all control positive sites (18,873), we found that all the positions showed VAF>25% (**Supplementary Figure 9**). Together with the robustness of the germline variant calling process in the original cell line sequencing[2], we are convinced that all the FN calls were truly existing variants in the source of the benchmark dataset (cell line mixtures).

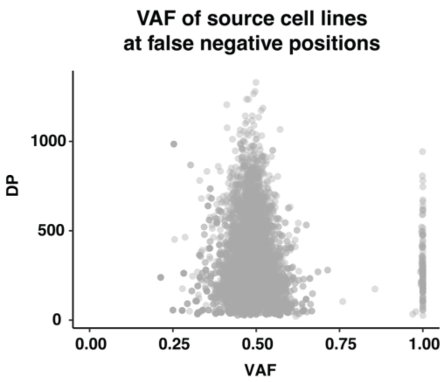

Supplementary Figure 9. Distribution of variant allele frequency of germline variants in source cell lines of ground truth. The VAFs in source cell lines are shown with sequencing depths at 18,873 control positive sites in 1,100× truth set.

Then, we found that sequencing data of the cell line mixtures may not contain the alternative alleles, even if the original cell line had, particularly when only a small portion of DNA was mixed to generate low VAF variants. For instance, we found that 2% and 17% of the true positive sites had no alternative alleles for 1% and 0.5% mixtures, in 1,100x coverage. It is true that no tools can call any true variants within these sites theoretically. Although undetectable, the final performance should include these FNs to represent the real-world level interpretation, because the same issue will be reproduced for all real analyses. Therefore, we calculated the theoretical upper limits of the calling performance for all evaluations of sensitivity and F1-score, to separate the FNs originated by miscalling from ones that are theoretically undetectable (**Supplementary Figure 10**).

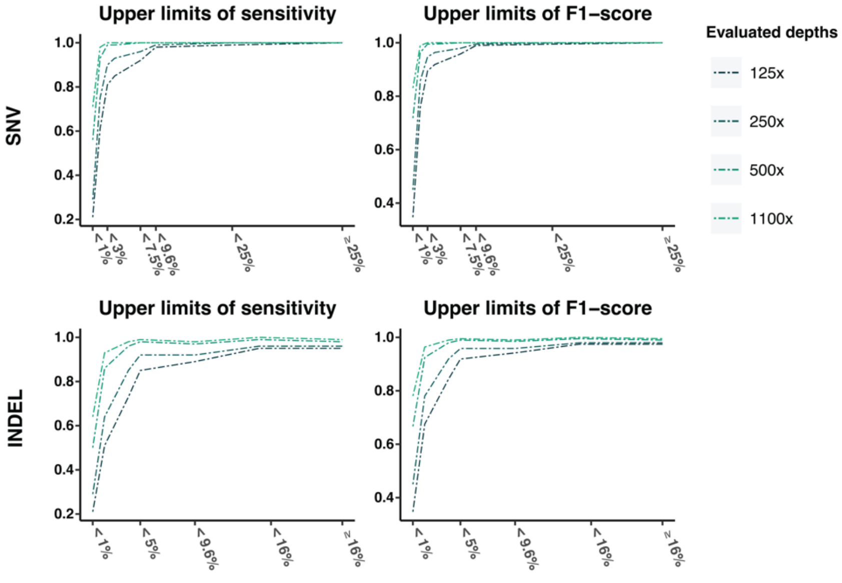

Supplementary Figure 10. Theoretical upper limits of sensitivity and F1-score. The upper limits depict the proportions of position with one or more alternative allele-containing reads at the positive control sites. Data shown were calculated with 39 truth sets in four different depths (125×, 250, × 500×, and 1,100×) according to VAFs, for both SNV and INDELs.

[3] Generation of the *in silico* simulation dataset

We generated in silico reference data set by computational simulation to compare with the biologically generated ground truth data utilized in this study. The *in silico* mixture dataset is another version of the ground truth variant set that was generated by mixing BAM files (instead of DNA) of the six cell lines. Like in the biological reference standard, we generated 39 in silico mixtures with the same designated proportions (**Supplementary Table 1**). This would be a much simpler and cost-effective procedure than the mixing DNAs, because we do not conduct actual sequencing of the 39 samples. Also, it is much more convenient to achieve accurate target mixture-proportion.

However, when we assessed the error profiles from the *in silico* mixtures, we found that the error profiles (e.g., the positions and types of the sequencing errors) are confined to the ones in the original cell line sequencing, which greatly limit the variety of the noises (**Supplementary Figure 11**). Consequently, the overall evaluation cannot be conducted with sufficient noise sources and levels that we face in the real-world settings. Therefore, we concluded that the mixture-derived reference standard is a more appropriate approach for a mosaic variant detection benchmark study.

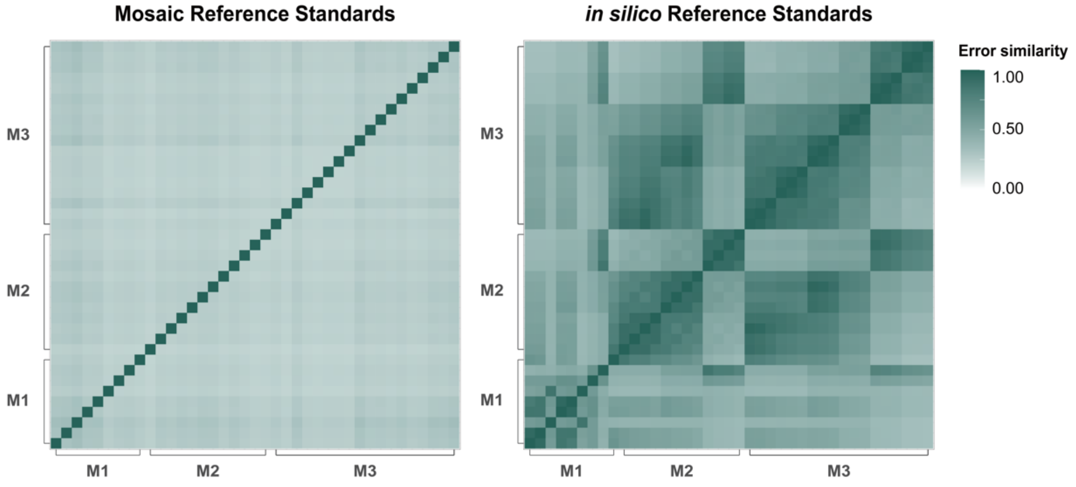

Supplementary Figure 11. Comparison of error profile in truth sets (a) Comparison of the error profiles between the mosaic reference standards and in silico reference standards with 39 truth sets. The *in silico* reference standards were generated with the identical combinations and mixing ratios. Unexpected alternative alleles in the non-variant negative control sites were calculated with the sequence data pileups and the similarities of them were calculated by Jaccard Index. M1, M2, and M3 refers to the three different categories of the reference standards depending on the mixing combination.

Methods to generate in silico dataset: We collected the BAM files of the original source cell lines from Sequence Read Archive under the accession code [PRJNA758606] and used Picard (v.2.2.2) DownsampleSam for down sampling and Samtools (v.1.14) merge for merging the BAM files. After generating the down-sampled BAM files according to the mixing ratios and combinations, 39 in silico reference dataset were generated.

To compare the error profiles in the reference data to the *in silico* simulation data, we generated pileups (Samtools mpileup v.1.14) for both data sets to gather the raw allele counts with default parameter settings. The unexpected alternative allele counts in each data were then collected based on the reference homozygous negative control sites, if there was one or more alternative alleles on the negative control sites. After that, the Jaccard similarity of the errors in each data were compared to all the other samples in pair-wise manner, resulting in 741 cases by selecting two out of 39 samples.

[4] Defining the relative distance between samples in a developmental lineage tree

We demonstrated a new strategy that utilize the relative distance in developmental hierarchy within multi (≥3) samples. By comparing the common ancestors in each pair of samples, we can define sample relatedness and their relative distance. First, we define the distance between a sample pair p1 = (A, B) in a phylogenetic tree using their most recent common ancestor c(A, B) (**Supplementary Figure 12**). We say a sample pair p1 = (A, B) is relatively proximal to a sample pair p2 = (A, C), if c(p2) = c(p1, p2) and c(p1) ≠ c(p1, p2). In other words, a sample pair is in a more proximal lineage if they are branched out more recently. For example, two samples developed from same germ layer (e.g., brain and skin; both from ectoderm) are more proximal than ones from two different germ layers (e.g., brain and skeletal muscle; ectoderm and mesoderm). Likewise, the relative distance of the samples in three mosaic types (M1-M3) could be defined, wherein samples in a more proximal lineage indeed had more shared variants. Although the cellular heterogeneity in tissue (e.g., blood in tissue) can limit the clarification of the origin of each tissue in real-world, using a subset of well-characterized somatic mutations could be utilized for reconstructing the developmental lineages.

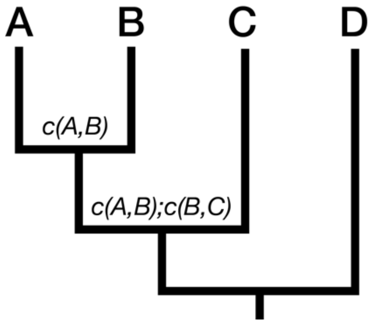

Supplementary Figure 12. Diagram to define the relative distance between samples. c(Sample1, Sample2) denotes the common ancestor between Sample1 and Sample2.

[5] The effect of capture bias in the exome-based benchmark result

To investigate the effect of the capture bias on the performance evaluation, we first calculated the sequencing depths at the positive control positions of SNVs and INDELS in the 39 ground truth data (Samtools mpileup v.1.14). The average sequencing depths was 1,100× calculated by the Qualimap (v.2.2.1), but the depths at the positive controls had high deviation and resulted lower average depths (616.2× and 139.0 for SNV and INDEL respectively). As a result, we confirmed the (1) variance towards genomic positions (2) consistent lower depth in INDEL sites (**Supplementary Figure 13 and Supplementary Table 6**). For INDELs, the fluctuation of the sequencing depths was observed as much high, showing 121× at the 1st quantile and 684× at the 3rd quantile (see table below). It is an intrinsic characteristic of capture-based whole exome sequencing that reflects the real-world performance which possibly affect overall accuracy.

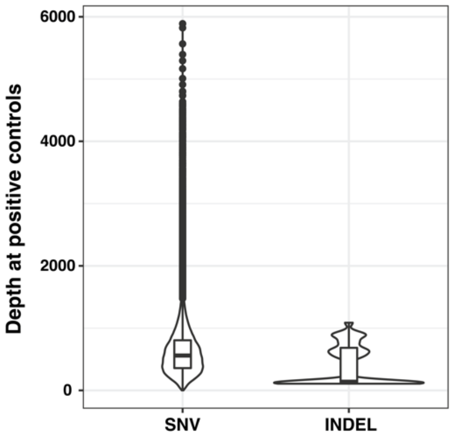

Supplementary Figure 13. Sequencing depths at SNV and INDEL positive controls in 1,100× exome data. The sequencing depths at all positive control SNVs and INDELs from 39 truth sets are shown. Boxplots with first and third quantiles with median are shown with whiskers representing maximum and minimum.

Depth at positive controls	SNV	INDEL
**1st quantile**	358.0	121.0
**2nd quantile (Median)**	560.0	139.0
**3rd quantile**	805.0	684.0
**Mean**	616.2	139.0

Supplementary Table 6. Sequencing depths at SNV and INDEL positive controls. Three quantiles and mean of sequencing depths at SNV and INDEL positive controls 39 truth set are shown (1,100×).

In addition, we also tested the extent of the coverage unevenness affecting the performance evaluation. We divided the ground truth data of SNVs into three subsets based on base coverage: lowest 25%, medium 50%, and highest 25%, which corresponded to <360×, 360× to 803×, and >803×, respectively. We then compared the sensitivity, precision, and F1-score between the three subsets, observing that the effect of coverage variability depends on the VAFs (**Supplementary Figure 14**). For the low VAF variants (< 10%), the overall performance (F1-score, rightmost column) was higher in the high coverage regions. In contrast, we did not observe a significant effect in the medium and high VAF variants. Overall, it is true that the mosaic variant calling performance is variable within the exon regions in the same sequencing data, but the effect is limited to low-VAF variants.

In the same context, we expect that the reported variant calling performance will be similar or slightly higher in the whole genome- or panel-sequencing if subjected to the same target depth. Because the coverages are more uniform in WGS for both genome and in panel-seq for targeted intervals, which reduces the portion of low-coverage regions, compared to WES (**Supplementary Figure 15**). Therefore, assuming the same target depth, a slight increase in performance is expected in genome or panel-seq.

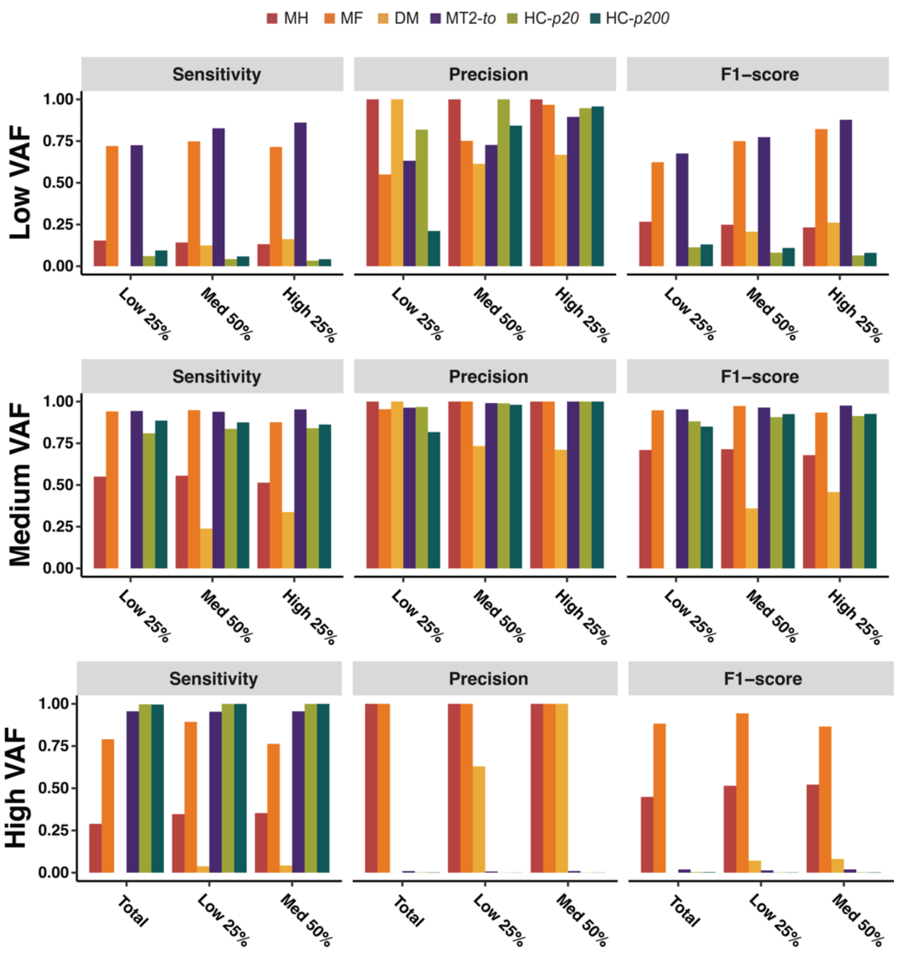

Supplementary Figure 14. Performance comparison with read depth variabilities in exome sequencing data. The 39 truth sets were divided into three subsets based on depths, low 25%, medium 50%, and high 25% (to <360×, 360× to 803×, and >803×). Sensitivity, precision, and F1-scores were calculated on three different VAFs: low (<10%), medium (10%<VAF<25%), and high (>25%).

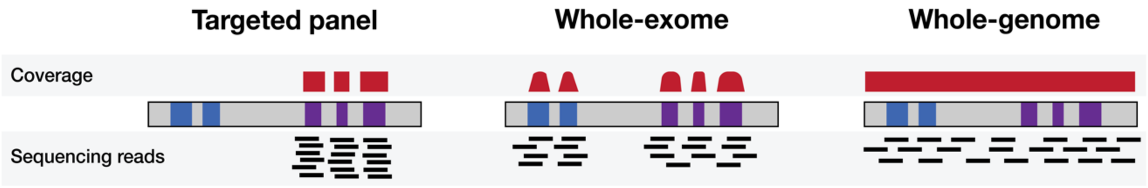

Supplementary Figure 15. Coverage consistency in sequencing data targeting different size of the genomic region.

[6] Possible noise in the ground truth data

To investigate the effect of noises due to the mixing procedure, we gathered the germline variants of the six cell lines, MRC5, RPE, CCD-18co, HBEC30-KT, THLE-2, and FHC. The merged number of germline variants was 62,719 in the target region of the whole exome sequencing (SureSelect Human All Exon V6, Agilent Technologies, Inc., CA, USA). We calculated the distances between all positive controls (18,873) and the nearest germline variants in source cell lines and observed the average 1,772 bp (**Supplementary Figure 16**). Despite the average distance was large, we found that approximately 39% of the true variants were accompanied with germline variants within a read length (150 bp). Even though mutations can exist with germline or subclonal mutations within a read[3], we acknowledge a potential over-representation that comes from multiple cell lines.

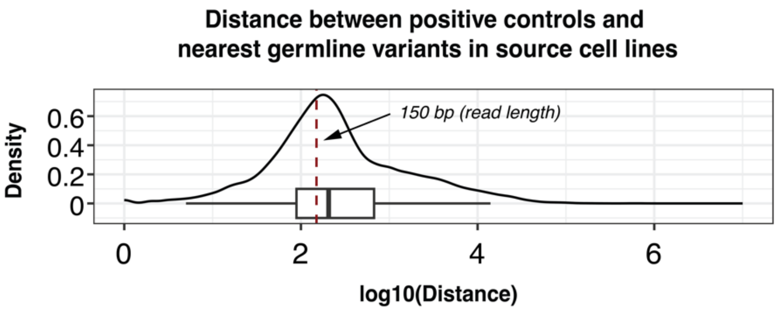

Supplementary Figure 16. Distance between positive controls and nearest germline variants in source cell lines. The densities of the log 10-scaled distance were calculated from the 39 truth sets. Three quantiles are shown with boxplot with the minimum and maximum value as whiskers. The red dashed line depicts 150 bp which is the length of the sequencing reads utilized in this study.

To systematically measure the potential effect, we calculated the sensitivities of all tools for only variants with proximal (<150 bp) germline variants. In all tools except MT2 and MF, no significant differences were observed. In contrast, sensitivity was approximately 4.9% and 4.1% lower in MT2 and MF (see **Supplementary Table 7**), implying a potential negative effect on these tools; we assume that the "clustered event" filtration strategy of MT2 makes these tools more sensitive to the presence of nearby germline variants. Again, 9-44% (27% in overall) of subclonal variants were observed with nearby germline variants[3], and the same decrease in sensitivity would be reproduced in real-world data. So, we assume the true effect from our study design should be partial. Also, the overall conclusion and the final best practice have not been affected because MT2 and MF were already top ranked with significant gaps from the others.

Tools	Sensitivity (all)	Sensitivity (germline variants within 150 bp)	Difference
MH	0.2001	0.1959	0.0042
**MT2**	**0.8402**	**0.7909**	**0.0493**
HC20	0.2322	0.2382	-0.0060
DM	0.1025	0.0982	0.0042
**MF**	**0.7624**	**0.7211**	**0.0412**
HC200	0.2510	0.2578	-0.0068

Supplementary Table 7. Difference of sensitivities with positive controls that are proximal to germline variants. A subset of positive controls from 39 truth sets were selected when one resides with proximal germline variants within a read. The sensitivities of the subset were compared to the original sensitivities and the differences of them are shown.

[7] The impact of the populational frequency database utilization with germline variant-derived ground truth data

When evaluating tools that utilize populational frequency databases for filtering germline variants (MosaicHunter, DeepMosaic, and Mutect2), we had to exclude the entries of positive controls from those databases whenever they existed. The purpose of this modified application was to prevent each tool from treating the true positive variants as known SNP positions. By doing this, we assumed that germline variant-derived true variants are equivalent to the sporadically generated variants regarding measuring variant calling accuracy.

To investigate if this modification introduced bias to the benchmark result, we conducted the same benchmark analysis on a subset of the true variants that are not located in SNP sites: the source of this non-SNP true variants is the rare germline variants in the original cell lines. We built three subsets from each database ("panel_of_normal": 1000g_pon.hg38.vcf.gz by Broad Institute, gnomAD: "hg38_gnomad211_gnomad.txt, and dbSNP build 154), resulting in to contain 53%, 5%, and 0.8% of the original call set respectively. We found that the evaluated performance result is almost same (**Supplementary Figure 17**), except a few intervals with small data points, which proves that the germline-derived construction did not lead to a bias.

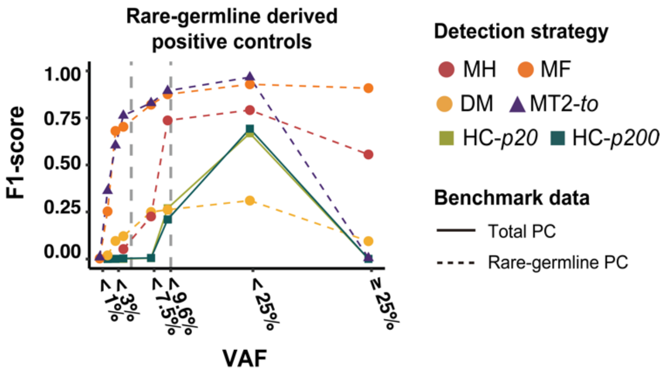

Supplementary Figure 17. Performance evaluation with non-SNP originated truth set. Three subsets of the positive controls where SNP-originated true variants were removed from the three populational frequency databases were used for evaluation. F1-scores of SNV performance are shown in the eight VAF bins (<1%, 1%–2%, 2%–3%, 3%–4%, 4%–7.5%, 7.5%–9.6%, 9.6%–25%, and ≥ 25%) with 1,100× data. PC, positive control

Moreover, we assessed if the restriction of the true variant positions to the non-SNP sites leads to another type of bias: mosaic variants that occur in known SNP sites have chances to be filtered out. This may lead to overestimated sensitivity as our true variant sets do not allow such filtration. However, we also assumed that the actual effect of the filtration is limited due to the rarity of the SNP sites of sufficiently high pAF (e.g., pAF > 0.001) compared to the genome size. To assess the amount of the possibly affected SNPs, we retrieved the sizes of the three pAF databases ("panel_of_normal", gnomAD, and dbSNP in same version

used in the benchmark) by their allele frequencies (**Supplementary Table 8**). We found that most of the entries were rare SNPs (pAF < 0.001). In total, 98,481,895 dbSNP and 51,447,633 gnomAD entries had pAF>0.001. Thereby, we could calculate the probability that a random mosaic variant hits these sites as 3.1% and 1.6% (numbers divided by 3.2B). In other words, even without allowing SNP regions, our benchmark dataset emulates > ~97% of real-world cases, which makes the overall result reliable.

We further assume that the true effect is even smaller because (1) not all the SNPs > 0.001 is filtered out (MosaicForecast uses the information as an optional post-filter, other tools use it as rather one of the supportive factors within classification models (DeepMosaic) or in calculation of mosaic probabilities (MosaicHunter)), along with more directly evident features from sequencing data. Also, (2) the actual match is even rarer; tools consider the ref-alt allele pair for match, which reduce the actual match by ~1/3. For PoN, all the matches are filtered regardless of the pAFs and the matching probability was 0.08% (2,609,566 divided by 3.2B).

pAF range	# dbSNP entries (%)	# gnomAD entries (%)	# PoN entries
**0-0.000001**	9,964,158 (1.69%)	98,698,804 (12.14%)	
**0.00001-0.000001**	165,550,421 (28.15%)	339,141,353 (41.71%)	
**0.0001-0.00001**	195,698,500 (33.28%)	264,212,535 (32.50%)	
**0.001-0.0001**	118,355,282 (20.13%)	59,552,322 (7.32%)	2,609,566
**0.001-0.01**	44,086,369 (7.50%)	27,301,443 (3.36%)	
**0.01-0.1**	8,094,187 (1.38%)	14,265,332 (1.75%)	
**0.1-1**	46,301,339 (7.87%)	9,880,858 (1.22%)	
**Total**	588,050,256 (100%)	813,052,647 (100%)	2,609,566 (100%)
**Total (pAF < 0.001)**	98,481,895 (16.7%)	51,447,633 (6.32%)	-

Supplementary Table 8. Proportion of the positive controls matching the entries in populational allele frequency databases. The proportions of the positive controls in 39 truth sets that matched with the three populational allele frequency databases, dbSNP, gnomAD, and panel of normal (**Methods**) utilized by MosaicHunter, MosaicForecast, and Mutect2 are shown.

[8] Performance evaluation with biological datasets and confirmation of the suggested recommendation

We conducted performance evaluation of mosaic variant detection methods with additional independent biological datasets, to confirm reproducibility of our benchmark results and the recommendations (**Table 1**). Three independent biological datasets were selected, in which the answer sets were validated with orthogonal experiments. Importantly, utilizing multiple datasets was imperative, because the features of mosaic variants (e.g., variant allele frequency (VAF) and variant-sharing) and the optimal detection methods can be largely varied. Thus, we selected three previously published mosaicism studies that had clearly validated answers, originated from different tissues within various study designs. First, we obtained deep sequenced (~500X) multi-organ WES data (Kim et al. PLOS Genetics 2022, **BioData1**)[4]**,** which provided 130 validated positions with 117 of them validated as true, where majority of them were shared by two or more organs. Also, we utilized 250X WGS data of sperm and blood pairs from eight individuals (Breuss et al. Nature Medicine 2020, **BioData2**)[5], 57 true shared mosaic variants. Lastly, we acquired 250X WGS data from a single neurotypical brain tissue followed by robust and extensive validation experiments, conducted by the Brain Somatic Mosaicism Network (BSMN) (Wang et al. Genome Biology 2021, **BioData3**)[1]. From this data, we could obtain 43 true positives with 357 false positives derived from various error sources. The detailed information of the three studies and their answer sets are described in the **Supplementary Table 9**.

	Original study	Data type	Tissue	Original detection method	Validation method	# Validated mutation		# Low VAF (<10%)		# Medium VAF (10%-25%)		# High VAF (> 25%)	
**BioData1**	**Kim et al. 2022 Plos Genetics**	~500 x WES	• Brain<br>• Heart<br>• Liver	• Mutect2<br>• RePlow<br>• NeuSomatic	Deep-targeted amplicon or Sanger sequencing	**130**		89		27		14	
						**True**	**False**	True	False	True	False	True	False
						**117**	**13**	78	11	26	1	13	1
**BioData2**	**Breuss et al. 2020 Nature Medicine**	200x WGS	• Sperm<br>• Blood	• Triodenovo with trio data	Targeted amplicon sequencing	**132**		102		25		5	
						**True**	**False**	True	False	True	False	True	False
						**114**	**18**	84	18	25	0	5	0
**BioData3**	**Wang et al. 2021 Genome Biology**	250X WGS	• Brain	6 different analytic methods including<br>• MosaicHunter<br>• Mutect2<br>• Strelka2<br>• HaplotypeCaller ploidy 2-10	PCR amplicon-based deep sequencing validation and multiplex PCR-based targeted single-end resequencing assay	**400**		359		18		23	
						**True**	**False**	True	False	True	False	True	False
						**43**	**357**	37	322	4	14	2	21

Supplementary Table 9. Independent biological datasets utilized for evaluation. Data types, tissues, original detection and validation methods, and number of the true and false answer sets are shown.

(1) VAF landscapes of the mosaic variants in the ground truth and the biological datasets

> **Recommendation #1**: Mosaic variant calling is not a task of a single kind, but a set of diverse problems, whose characteristics and difficulties vary largely. The number of samples, presence of matched controls, VAFs, and variant-sharing patterns are major factors that affect the algorithmic performance and should be carefully considered before analysis.

In the same context with our **Recommendation #1**, the characteristics of mosaic variants can be largely varied under each study design, which can directly affect the detection performance. Among all mosaic variant features, variant allele frequency (VAF) was one of the most critical factors that can alter detection strategy. Thus, we first investigated the VAF distributions of the mosaic variants in this study (ground truth data) and those in five independent mosaicism studies[1, 4-7], including three of them utilized for the additional evaluation (BioData 1-3). As expected, the VAF distributions were observed to be highly dependent on the tissues that were sequenced (**Supplementary Figure 18**). For example, mosaic variants from Breuss et al. 2022 (study in brain) were mostly enriched in low VAF (<5%), whereas ones from Hsieh et al. 2020 (study in heart) were distributed in a wider VAF range (5-25%). The only common characteristic was the general enrichment in low VAFs and the rapid decrease in the number of high VAFs. Since our benchmark covers diverse conditions and scenarios in mosaic variant calling, we selected the three biological datasets BioData1-3 (Kim et al. 2022, Breuss et al. 2020, and Wang et al. 2021) that were previously mentioned. In this way, we could secure not only the validated variants with varied range of VAFs but also covering variants in various scenarios such as balanced or imbalanced shared variants and sample-specific variants.

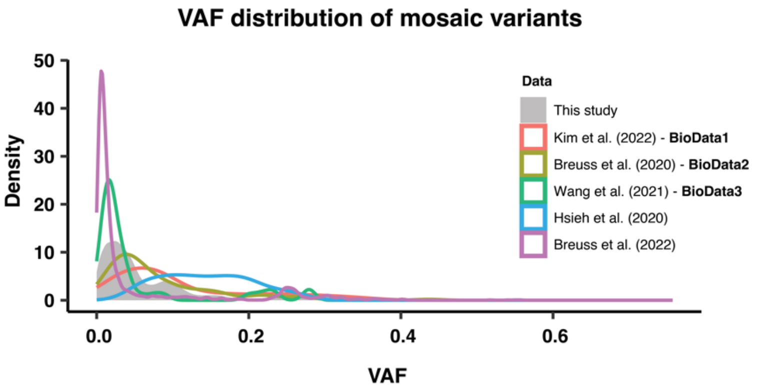

Supplementary Figure 18. The distribution of variant allele frequencies of true mosaic variants in the ground truth data and five other biological studies.

(2) Mosaic variant detection in a single sample

> **Recommendation #2:** MosaicForecast and Mutect2 are generally recommended for calling mosaic SNVs in a single sample. These tools are particularly strong in calling low-VAF mutations (<10%).
>
> **Recommendation #3:** MosaicHunter showed low sensitivity but extremely high precision. Together with other callers, these calls can be used for strict filtering or prioritization.
>
> **Recommendation #5**: Call set concordance between callers and read depths is very low. Therefore, finding overlaps from different callers to achieve high confidence is not recommended. The use of multiple callers should be composed in a way of assigning one to the best-performing VAF area.

In single sample mosaic calling, we recommended MosaicForecast (MF) and Mutect2 tumor only mode (MT2-*to*) to achieve the best performance (**Recommendation #2**). We once more confirmed that both showed superb performance among the six single sample detection methods (MH, MF, DM, MT2-*to*, HC-*p20*, and HC-*p200*) in all three datasets (**Supplementary Figure 19**). Indeed, their performance at low VAF (<10%) were clearly the best. Importantly, the answer sets of BioData1 and BioData2 highly lacked negative controls compared to real-world data, because they were often collected with multiple callers to generate high-precision call set for the experimental validations. Resultantly, except for the BioData3 which was informative owing to enough negative controls (89%), the precision of the BioData1 and BioData2 reached almost the maximum (1) regardless of the methods. It is noteworthy that the precision obtained from BioData3 was shown to be greatly consistent with our benchmark result, in which MF and MH had the highest precision in overall VAFs while the somatic and germline callers (MT2-*to*, HC) showed a rapid decrease in precision at high VAF (>25%) area. We also confirmed that MH showed low sensitivity but extremely high precision (**Recommendation #3**). The call set concordance between callers was again confirmed to be low and it is not appropriate to take an intersection of multiple callers to achieve a high confidence call set (**Recommendation #5, Supplementary Figure 20**).

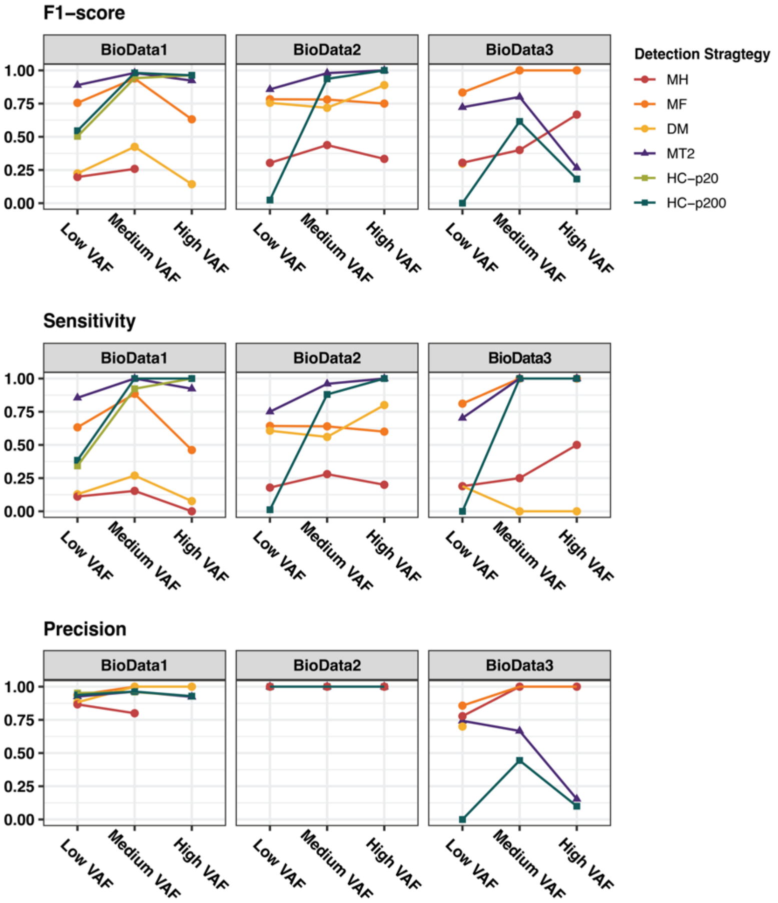

Supplementary Figure 19. Performance evaluation of single sample mosaic calling with biological datasets. F1-score, sensitivity, and precision are shown in three VAF ranges: low (<10%), medium (10-25%), and high (>25%). Precision and F1-score could not be obtained when the number of true and false positives were both zero.

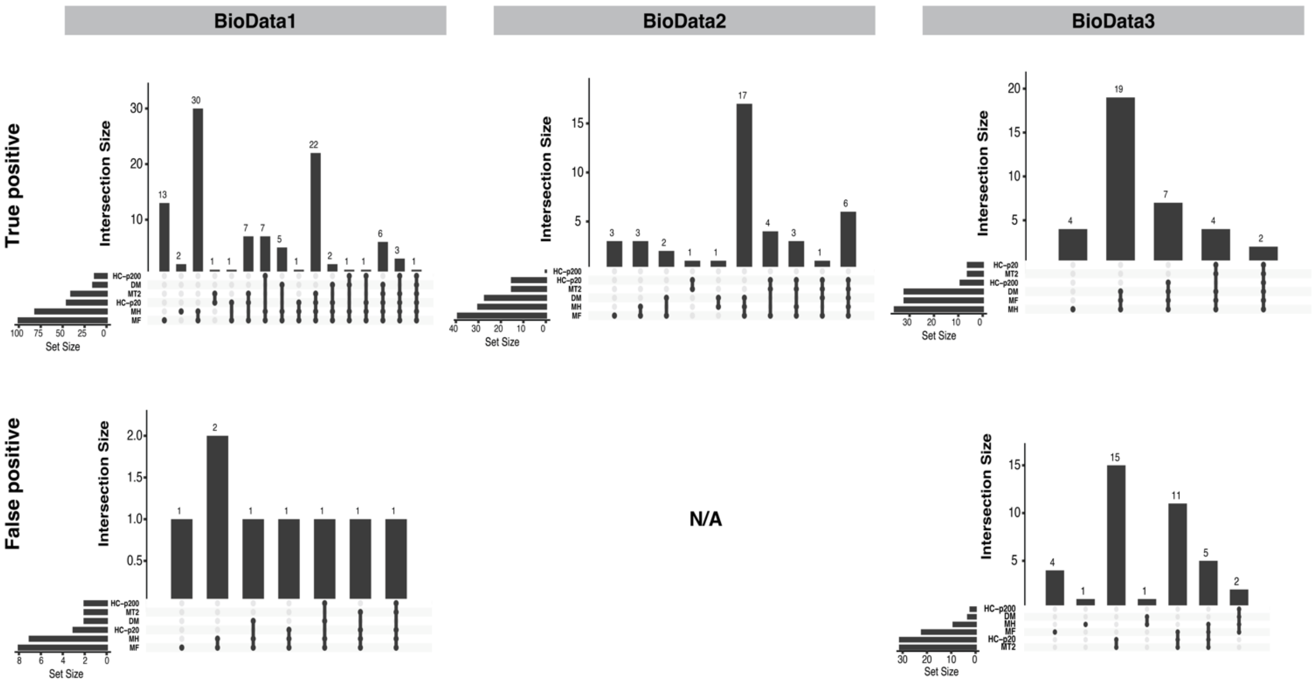

Supplementary Figure 20. Call set concordance between detection methods in the biological data. The true and false positives from the three biological datasets (Kim et al. 2022, Breuss et al. 2020, and Wang et al. 2021) were gathered after applied to the six single sample calling methods and intersected. UpSetR (1.4.0) was used to generate the figure.

(3) Call set and feature level recombination in mosaic calling

> **Recommendation #12:** Provided with numerous features, scores and filters from multiple algorithms, a complementary ensemble approach can be an efficient start to improve performance without developing a new algorithm from scratch.

In this benchmark, we demonstrated the three examples of the feature-level recombination within the tested algorithms, showing that informative features from one algorithm can bring a complementary effect to another one (**Recommendation #12**). Here, we tested the three examples that were demonstrated with the ground truth data. Since the answer sets from the biological data greatly lacked the negative controls, the ratio of true and false positives in BioData1 was 12.5. Moreover, there was no false positives in BioData2, so couldn't be assessed. However, the BioData3 could be properly applied as it had abundant false positives, and we observed that 27% of the false positives from MT2-*to* call set could be removed when filtered by a MF feature (alt soft clip > 0.05), with only 3% loss of the true positives (see **Supplementary Table 10**). Also, 18% of the false positives from HC-*p200* call set could be removed without losing any true call. The false positives in precision-focused dataset (BioData1) couldn't be removed, and even small proportion of the true positives (1-6%) were removed, we expect that this kind of feature-level strategy would be more feasible at the variant calling steps, where various false positives threaten accuracy of the call sets.

Data	Ratio of #TP and #FP	Original Call	Feature used for filtration	Removed true positives (removed/total)	Removed false positives (removed/total)	% Loss of True Positives	% Removed False positives
**BioData1[#]**	**12.5**	**MT2-*to*[#]**	**MF alt soft clip > 0.05**	1/100	0/8	**1%**	**0%**
		**HC-p200**	**MT2-to MFRL alt < 150**	6/100	0/8	**6%**	**0%**
		**HC-p200**	**MF HetLH > 0.25**	0/100	0/8	**0%**	**0%**
**BioData3**	**1.5**	**MT2-*to*[#]**	**MF alt soft clip > 0.05**	1/32	6/22	**3%**	**27%**
		**HC-p200**	**MT2-to MFRL alt < 150**	0/32	0/22	**0%**	**0%**
		**HC-p200**	**MF HetLH > 0.25**	0/32	4/22	**0%**	**18%**
[#]high precision call set (ratio of #TP and #FP = 12.5)							

Supplementary Table 10. Feature-level recombination with biological data.

(4) Sample-specific mosaic variant detection in paired-sample

Recommendation #6: For sample-specific calling, current somatic callers (Mutect2 and Strelka2) outperform in overall VAFs including low VAF (<5%)

Among the three biological datasets, we could attain sample-specific variants with its validation results from BioData1, where the VAFs of the 25 true variants were distributed evenly from low (<10%) to high (>25%) (**Supplementary Table 11**). When an individual had more than two samples, the nine evaluated detection methods were applied to all possible sample pairs and only the variants existed in a single sample (not in all others) were collected. As a result, we confirmed that MT2 and STK2 showed the best F1-score across all VAF ranges including low VAF (<10%), as suggested in **Recommendation #6** (**Supplementary Figure 21**). In other words, somatic methods that utilize joint genotyping supported by matched controls are currently the best strategy for sample-specific mosaic variant calling.

Data	#Sample-specific mutations					
**BioData1**	Total					
	28					
	True			False		
	25			3		
	# Low VAF (<10%)	# Medium VAF (10%-25%)	# High VAF (> 25%)	# Low VAF (<10%)	# Medium VAF (10%-25%)	# High VAF (> 25%)
	7	9	9	2	0	1

Supplementary Table 11. Number and variant allele frequencies of sample-specific variant answer set. The number of sample-specific true and false answers across various variant allele frequencies are shown from BioData1.

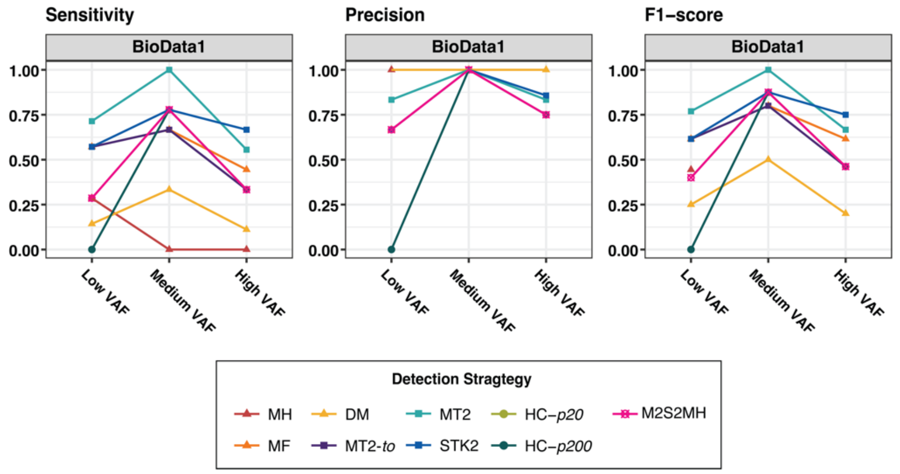

Supplementary Figure 21. Performance evaluation of sample-specific variant calling in biological dataset. Sensitivity, precision, and F1-score are shown in three VAF ranges: low (<10%), medium (10-25%), and high (>25%). A sample-specific variants were collected from BioData1 if they were called in all possible combinations of sample pairs when three or more sample existed in an individual.

(5) Shared mosaic variant detection in paired-sample

Recommendation #7: For shared variant detection, although MosaicForecast and Mutect2 showed good overall accuracy, we want to highlight that the best-performing tool for each VAF range varied. We recommend also trying out the best tools for the expected VAF-pair area if one exists.

To evaluate shared variant detection performance with biological datasets, we could apply BioData1 and BioData2, supported by 56 and 28 answer variants respectively. The answer sets were comprised of true variants that were shared by two to six samples from an individual (**Supplementary Table 12**).

<table>
<tr><th>Data</th><th colspan="8"># Shared mutation</th></tr>
<tr><td rowspan="6">BioData1</td><td colspan="8">Total</td></tr>
<tr><td colspan="8">56</td></tr>
<tr><td colspan="5">True</td><td colspan="3">False</td></tr>
<tr><td colspan="5">51</td><td colspan="3">5</td></tr>
<tr><td># Shared tissue</td><td>2</td><td>3</td><td>4</td><td>6</td><td># Shared tissue</td><td>2</td><td>3</td></tr>
<tr><td># Mutation</td><td>18</td><td>2</td><td>2</td><td>1</td><td># Mutation</td><td>1</td><td>1</td></tr>
<tr><td rowspan="6">BioData2</td><td colspan="8">Total</td></tr>
<tr><td colspan="8">28</td></tr>
<tr><td colspan="5">True</td><td colspan="3">False</td></tr>
<tr><td colspan="5">28</td><td colspan="3">0</td></tr>
<tr><td colspan="3"># Shared tissue</td><td colspan="2">2</td><td colspan="2"># Shared tissue</td><td>0</td></tr>
<tr><td colspan="3"># Mutation</td><td colspan="2">14</td><td colspan="2"># Mutation</td><td>0</td></tr>
</table>

Supplementary Table 12. Number of true and false shared variants in biological data.

We first generated VAF-VAF combinations of shared variants, where variants in each sample could be partitioned based on four different VAF ranges: very-low (VL): ≤ 5%, low (L): > 5% and ≤ 10%, medium (M): > 10% and ≤ 25%, and high (H): > 25%. After Partitioning the VAF ranges of a sample pair into 16 (= 4 × 4) areas and assigning the sample with higher VAF to the x-axis, we could obtain 10 blocks of VAF combinations. This was done for locating as many variants as possible in a single block to reduce bias during evaluation, because the number of the answer sets in biological data are highly limited. As a result, the true and false shared variants could be located within four (BioData1) and six (BioData2) blocks (out of ten) and utilized for evaluation (**Supplementary Figure 22**).

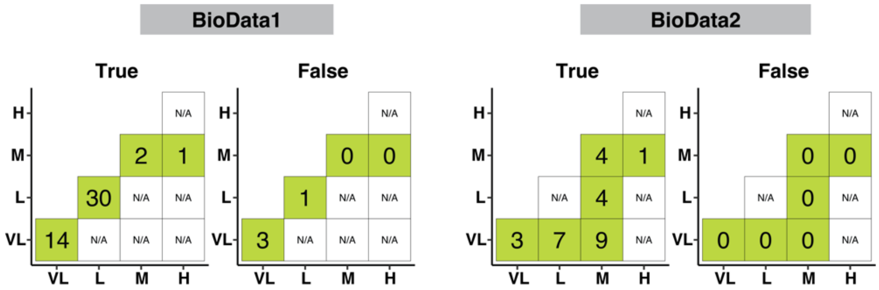

Supplementary Figure 22. VAF distribution of true and false shared variants in the biological datasets. The VAFs of samples having shared variants were partitioned into four VAF range groups: very low (VL), low (L), medium (M), and high (H) (<5%, ≥5% and <10%, ≥10% and <25%, and ≥ 25%, respectively). N/A=not evaluated (positive controls could not be assigned). A sample with higher VAF was located at x-axis.

After calculating the F1-scores for all possible VAF combination blocks, we confirmed that only MF and MT2 maintained good overall accuracy in both datasets (**Supplementary Figure 23**). Notably, several inevitable limitations existed while we validate the benchmark results with biological data, such as (1) the answer sets itself is already biased because it was often selected with one of the evaluated methods and (2) the answer set is highly precision-focused data which was conservatively chosen for validation experiments. For example, we observed MF showing both minimum (0) and maximum (1) F1-score at H-M (high-medium) VAF block in each dataset, owing to the lack of answer where only one true variant could be assigned in both datasets, without a false positive. Also, MH, M2S2MH (MH with rescue strategy) and DM were shown to have remarkably high performance in only one of the data (BioData2). We assume that this is because this data was used for the training of DM model construction and possibly MH could have affected the original call sets in some way. Nevertheless, good performance MF and MT2 were reproducible in both datasets, as suggested in **Recommendation #7**.

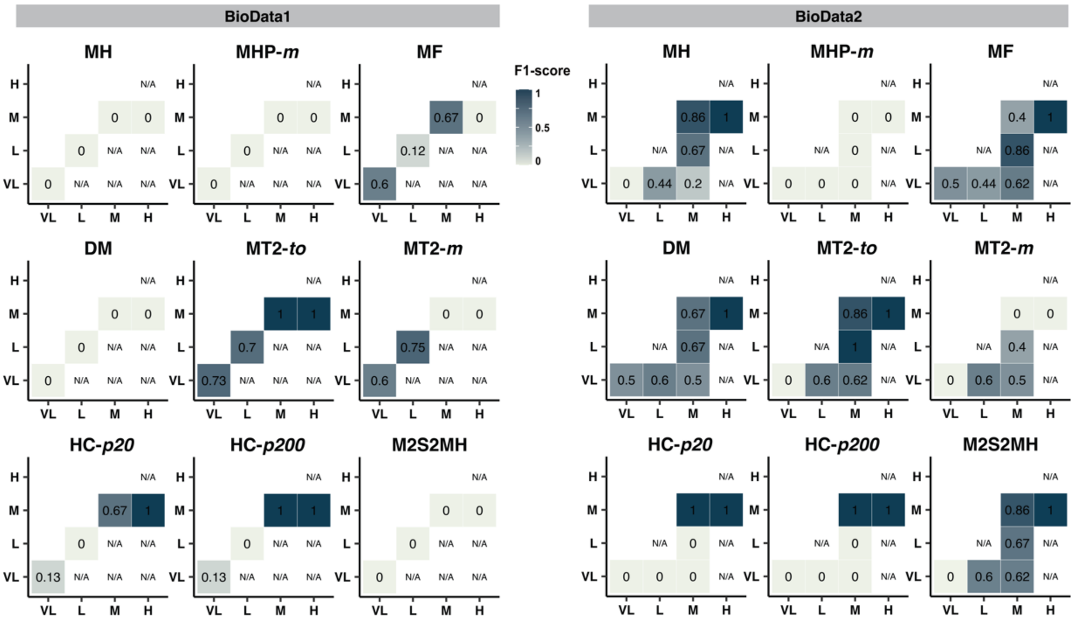

Supplementary Figure 23. Performance evaluation of shared variants in independent biological data. F1-scores of shared variant detections in ten possible VAF bin combinations are shown. Four VAF ranges (very-low (VL): ≤ 5%, low (L): > 5% and ≤ 10%, medium (M): > 10% and ≤ 25%, and high (H): ≥ 25%) were selected for analysis.

Moreover, we also suggested trying out the best tools for the expected VAF-pair area in shared variant detection, as the best-performing tool in each VAF combination area highly varies (**Recommendation #7**). In the main benchmark results, we noted as "MF was the best when both variants were present in a very low VAF (<5%), which is frequently the main target for brain mosaicism. In higher VAFs (>25%), germline approaches (HC-*p20* and HC-*p200*) showed the best performance (**Fig. 3f**)". Also, MT2-*to* marked the best performing tool in the rest of the VAF area (5%-25%).

Herein, in both biological datasets, we were able to reproduce the original combinations of the best performing-ensemble approach, with consideration of the three limitations in the biological datasets: (1) Mutect2 was used for the call set construction for the BioData1, (2) lack of true positives in some combination blocks (VL-VL and H-M), and (3) overestimated performance of MH and DM in BioData2 (**Supplementary Figure 24**).

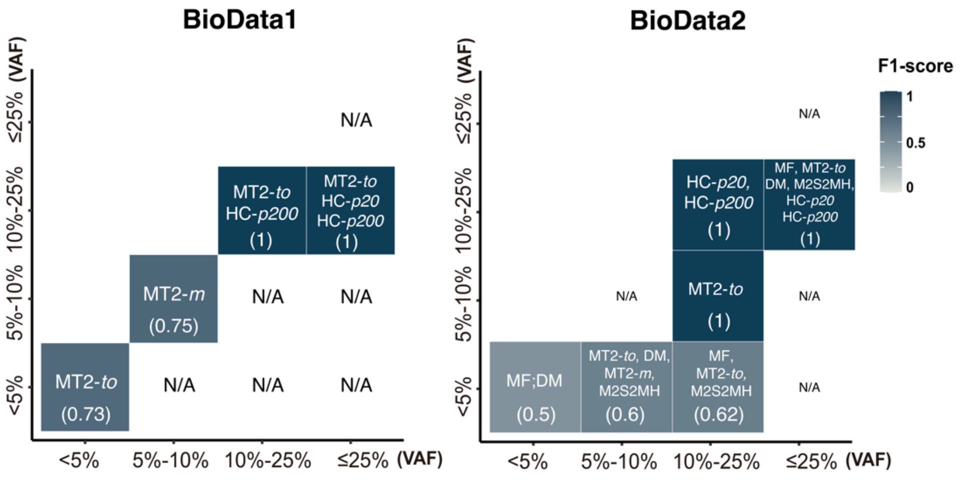

Supplementary Figure 24. An ensemble of the best performing tools in shared variant detection with biological data. Combinations of the best performing approaches within each combinational VAF area are shown. Each VAF combinatorial area was based on four VAF ranges (very-low (VL): ≤ 5%, low (L): > 5% and ≤ 10%, medium (M): > 10% and ≤ 25%, and high (H): ≥ 25%).

Lastly, we recommended using MF with a rescue strategy for shared variant detection (**Recommendation #8)**. The rescue strategy was suggested to improve the sensitivity of shared variant detection by rescuing the misclassified calls, calls that were detected in only one of the samples and missed in the other. As expected, we confirmed that MF with rescue strategy achieved a remarkable performance as a single method with BioData2, covering all the VAF areas including the most challenging VL-VL (very low in both, <5%) area (**Supplementary Figure 25)**. BioData1 could not be applied because no misclassified variants existed.

Recommendation #8: In the present situation, we recommend utilizing somatic callers (Mutect2 or Strelka2) for sample-specific variant and MosaicForecast with the rescue strategy for shared mosaic variant detection with paired samples

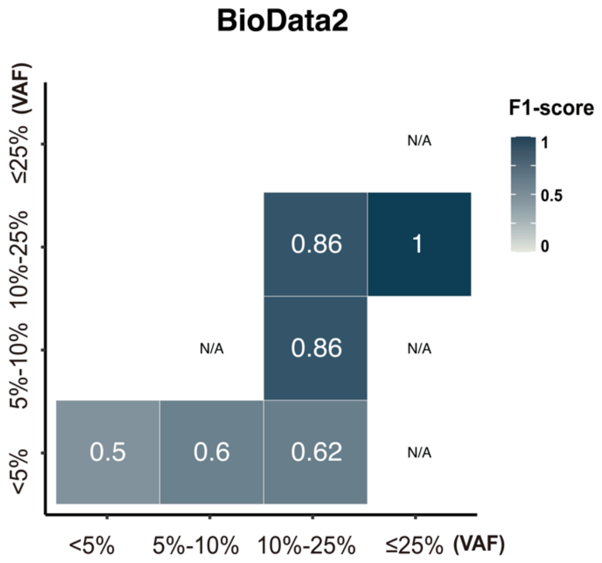

Supplementary Figure 25. F1-score of the MF with rescue strategy in shared variant detection. Each VAF combinatorial area was based on four VAF ranges (very-low (VL): ≤ 5%, low (L): > 5% and ≤ 10%, medium (M): > 10% and ≤ 25%, and high (H): ≥ 25%).

[9] Integrity of the cell lines utilized for the reference standard

The six immortalized normal cell lines (MRC5, RPE, CCD-18co, HBEC30-KT, THLE-2, FHC) utilized for the truth sets construction are described in a previously published paper[2]. Briefly, FHC(#CRL-1831) and THLE-2 (#CRL-2706) cells were purchased from the American Type Culture Collection (ATCC). RPE (#00194987) was purchased from Lonza Bioscience. MRC5 (#10171) and CCD- 18co (#21459) were purchased from the Korea Cell Line Bank. HBEC30-KT is a transformed cell line of HBEC with two genetic alterations (CDK4, hTERT)[8], and its genomic DNA is available under request. The STRs (short tandem repeats) of all cell lines except for RPE, were cross compared to the previously reported STRs to confirm the integrity and reproducibility. The STRs of the RPE were newly reported in the previous paper. All cell lines were verified to not have any mycoplasma contamination and none of them are reported in the International Cell Line Authentication Committee (https://iclac.org/databases/cross-contaminations).

[10] Selection of the raw input for MosaicForecast

The selection of Mutect2 raw call sets as an input of the MF was based on the recommendation by the authors[9]. As the authors also noted that other algorithms can be used, we tested and compared the MF performance with two different inputs, raw call sets of MT2 and HC-p200 (**Supplementary Figure 26**). We found two times more false positives in HC-p200 call sets in the low VAF (<10%) area, thereby resulting in much lower F1-score. We speculate that MF has been optimized with the MT2 input from its initial model training (with MT2 call sets) and recommend using MT2 raw calls when using MosaicForecast.

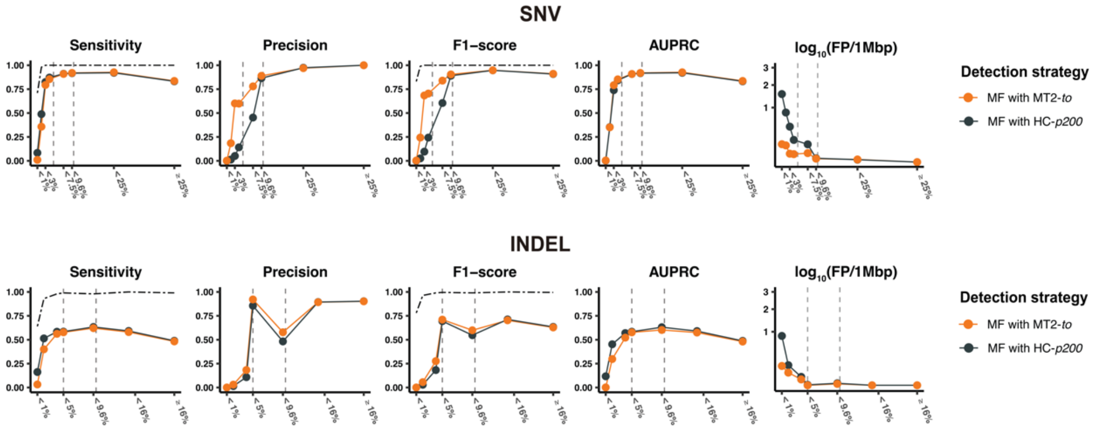

Supplementary Figure 26. Comparison of MosaicForecast performance with different input, Mutect2 and HaplotypeCaller with ploidy 200. Sensitivity, precision, F1-score, AUPRC, and the false positive rate (log_{10}FP/1Mbp) in different VAF bins categories are shown. Dashed lines with dots depict the upper limit for the true positives. The two vertical lines with gray dash refers to VAF 5% and 10%. The y-axis of log_{10}(FP rate) is shown in square root. SNV detection performance of six applied approaches in the eight VAF bins (<1%, 1%–2%, 2%–3%, 3%–4%, 4%–7.5%, 7.5%–9.6%, 9.6%–25%, and ≥ 25%) with 1,100× data. INDEL detection performance of four applied approaches in the seven VAF bins (<1%, 1%–2%, 2%–4%, 4%–5%, 5%–9.6%, 9.6%–16%, and ≥ 16%) with 1,100× data.

References

1. Wang, Y. et al. Comprehensive identification of somatic nucleotide variants in human brain tissue. *Genome Biol* **22**, 92 (2021).
2. Ha, Y.J. et al. Establishment of reference standards for multifaceted mosaic variant analysis. *Sci Data* **9**, 35 (2022).
3. Bohrson, C.L. et al. Linked-read analysis identifies mutations in single-cell DNA-sequencing data. *Nat Genet* **51**, 749-754 (2019).
4. Kim, J.H. et al. Analysis of low-level somatic mosaicism reveals stage and tissue-specific mutational features in human development. *PLoS Genet* **18**, e1010404 (2022).
5. Breuss, M.W. et al. Autism risk in offspring can be assessed through quantification of male sperm mosaicism. *Nat Med* **26**, 143-150 (2020).
6. Hsieh, A. et al. EM-mosaic detects mosaic point mutations that contribute to congenital heart disease. *Genome Med* **12**, 42 (2020).
7. Breuss, M.W. et al. Somatic mosaicism reveals clonal distributions of neocortical development. *Nature* **604**, 689-696 (2022).
8. Sato, M. et al. Human lung epithelial cells progressed to malignancy through specific oncogenic manipulations. *Mol Cancer Res* **11**, 638-650 (2013).
9. Dou, Y. et al. Accurate detection of mosaic variants in sequencing data without matched controls. *Nat Biotechnol* **38**, 314-319 (2020).
